# Inferring Social Network Structure from Bacterial Sequence Data

**DOI:** 10.1371/journal.pone.0022685

**Published:** 2011-08-01

**Authors:** Mateusz M. Pluciński, Richard Starfield, Rodrigo P. P. Almeida

**Affiliations:** 1 Department of Environmental Science, Policy and Management, University of California, Berkeley, California, United States of America; 2 Division of Epidemiology, School of Public Health, University of California, Berkeley, California, United States of America; Umeå University, Sweden

## Abstract

Using DNA sequence data from pathogens to infer transmission networks has traditionally been done in the context of epidemics and outbreaks. Sequence data could analogously be applied to cases of ubiquitous commensal bacteria; however, instead of inferring chains of transmission to track the spread of a pathogen, sequence data for bacteria circulating in an endemic equilibrium could be used to infer information about host contact networks. Here, we show—using simulated data—that multilocus DNA sequence data, based on multilocus sequence typing schemes (MLST), from isolates of commensal bacteria can be used to infer both local and global properties of the contact networks of the populations being sampled. Specifically, for MLST data simulated from small-world networks, the small world parameter controlling the degree of structure in the contact network can robustly be estimated. Moreover, we show that pairwise distances in the network—degrees of separation—correlate with genetic distances between isolates, so that how far apart two individuals in the network are can be inferred from MLST analysis of their commensal bacteria. This result has important consequences, and we show an example from epidemiology: how this result could be used to test for infectious origins of diseases of unknown etiology.

## Introduction

The widespread availability of DNA sequencing has led to their increased use as tools in the study of infectious disease dynamics. It has been used to track the spatiotemporal spread of pathogens and to infer chains of transmission for various bacteria and viruses, including HIV [1], MRSA [2], rabies [3], foot and mouth disease [4], hepatitis C [5], and tuberculosis [6]. These studies have as their primary focus the pathogen itself – the implicit goal of understanding disease dynamics is the eventual control of pathogen spread. Here, we argue that sequence data for ubiquitous commensal bacteria – an endemic instead of epidemic setting – can instead be used as a tool to study the host contact network. The structure of the host contact network is known to strongly affect the dynamics of infectious diseases [7]. Moreover, network structure also strongly determines the population genetics of the pathogen spreading on the network. For example, previous modeling studies have shown that the degree to which a network is randomly wired affects the overall diversity of strains of commensal bacteria such as *Neisseria meningitidis* [8] [9].

**Figure 1:**
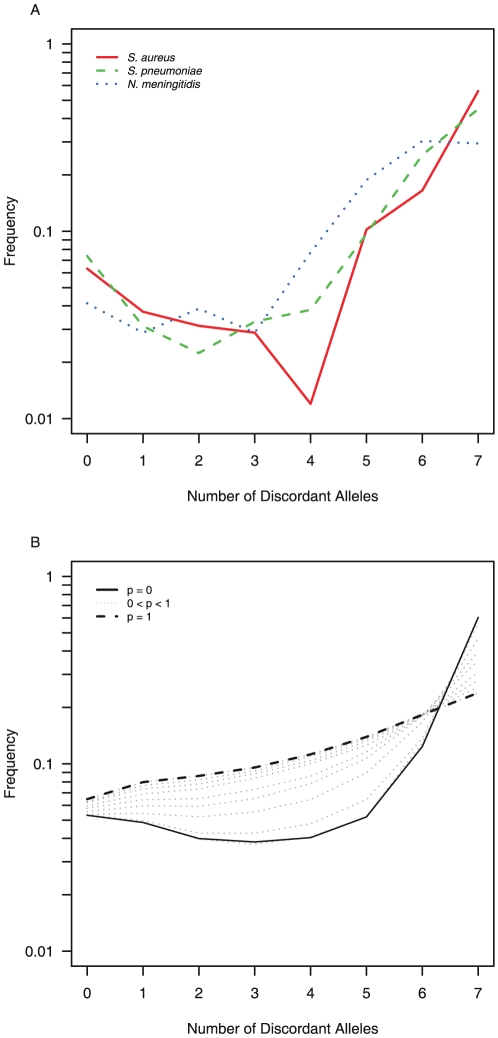
A) Observed distributions of pairwise number of discordant alleles for S. *aureus*, *N. meningitidis*, *S. pneumonia*. Data from [18] [19] [20], cited in [11]. B) Distributions of pairwise number of discordant alleles drawn from simulated networks with various values for the small world parameter 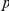.

Recently, multilocus sequence typing (MLST) has become one of the most popular techniques for the genotyping of bacteria, and involves the amplification and sequencing of several (usually seven) housekeeping genes, with a sequence type being defined by the combination of its seven alleles [10]. One way to summarize MLST data for isolates from a population is to calculate the distribution of pairwise distances, defined as the number of discordant alleles. For many commensal bacteria, including *Neisseria meningitidis*, *Staphylococcus aureus*, and *Streptococcus pneumoniae*, this distribution has a characteristic “U shape” (Figure 1A). This shape is inconsistent with traditional population genetics models of neutral evolution, which would predict either a strictly increasing or decreasing function [11]. In practice, the “U shape” is a result of an overrepresentation of clonal strains, and has been alternately attributed to small outbreaks of clonal strains (“microepidemics”) [11], or more recently, to heterogeneity in the reproductive potential of different strains under selective pressure from the host [12].

Fundamentally, the “microepidemic” explanation corrects for the overrepresentation of clonal strains by introducing an extra parameter to account for local spread. Here, we show that once network structure is accounted for, it is no longer necessary to explicitly account for this additional local spread - we show that certain network structures naturally lead to this characteristic “U shape”. Specifically, the degree of local structure in the network, defined by the small world parameter 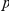 can be found to directly result in this characteristic shape. Moreover, if we assume that the network topology is the main determinant of the shape of the distribution of the number of discordant alleles, the shape of this curve can then be used to infer the structure of the host contact network.

## Results

A particularly robust way of modeling human social networks is to consider small world networks, networks that retain both the high clustering and low characteristic path length (meaning most points are separated by only a few nodes) characteristic of human networks [13]. Moreover, small world networks are parameterized in such a way that a single parameter, the small world parameter 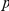, uniquely controls the global structure of the network, with 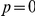 resulting in an ordered lattice-like network, and 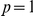 in fully random networks, and intermediate values of 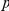 resulting in realistic small-world networks.

An individual-based model that simulates MLST data from commensal bacteria spreading among individuals linked together on a randomly generated small world network suggests that the characteristic “U shape” of the distribution of pairwise discordant alleles previously observed for commensal pathogens only occurs for some values of the small world parameter 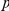 (Figure 1A). While the other parameters of the model do affect the form of the distribution (Figure S1), only the small world parameter controls the existence and magnitude of the dip in the distribution for intermediate values of the number of discordant alleles (Figure 1B).

Since the small world parameter is a measure of how structured the population is, in practice these results suggest that highly structured populations (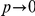) result in localized pockets of local strains, resulting in an overrepresentation of low discordance pairs – recent, local transmission – and maximally discordant pairwise comparisons between different pockets of local strains separated in the network. As the network gets more random (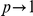), this local structure disappears, and the form of the distribution of pairwise discordant alleles becomes either strictly increasing or strictly decreasing, depending on the mutation rate (Fig. S1A).

**Figure 2:**
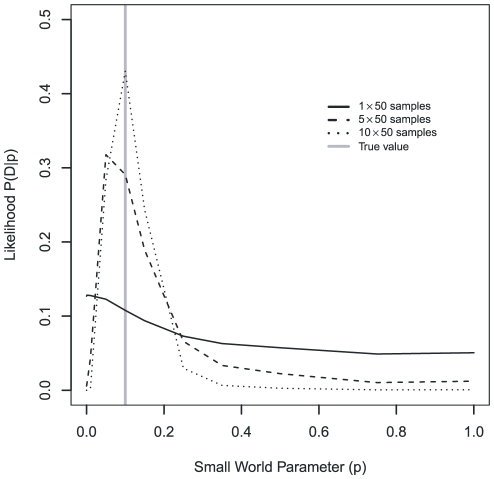
Likelihood of the small world parameter 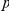, for increasing numbers of independent samples from networks with true small world parameter 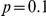.

Given that the small world parameter 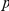 strongly determines the form of the pairwise genetic distance distribution, it seems plausible that given bacterial isolates sampled from a single population, one might be able to infer some information about the host contact structure of the population, specifically the small world parameter 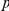. While the individual MLST datapoints are independent, the set of pairwise distances among them is not, and the likelihood consequently cannot be computed straightforwardly; we instead employ a variant of Approximate Bayesian Computation (see Methods). Using simulated MLST data from our model for a given random network with fixed small world parameter 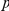, we ran inference on the set of pairwise distances. One sample of MLST data from 50 individuals resulted in a posterior distribution for 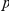 that peaked close to the true value, but whose uncertainty was quite wide. However, repeated independent samples of 50 isolates from the same kind of population at later times narrowed that peak (Figure 2). Our results therefore suggest that global properties of host contact networks, such as the degree of randomness, can indeed be inferred from MLST data for ubiquitous commensal bacteria spreading on that network.

**Figure 3:**
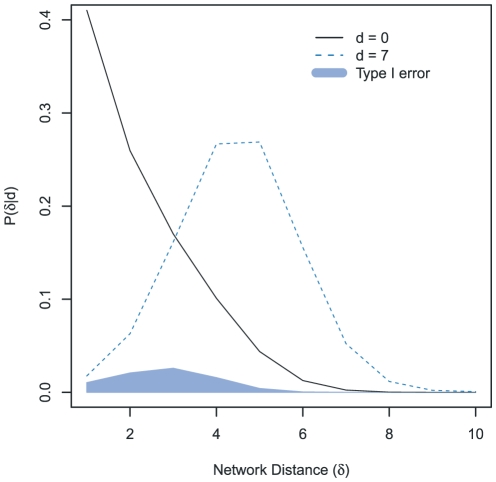
Likelihood for 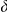, the number of nodes separating two individuals in the network, given that the number of discordant alleles in their isolates is 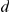 **.** The area of the shaded region is the probability that a pair of individuals with seven discordant alleles will be closer together in the network than a pair of individuals with no discordant alleles, the Type I error (see Methods, and Figure S3).

If global network properties can be inferred from MLST data then it is also plausible that some of the local network structure can also be gleaned from the same data. While it is not feasible to reconstruct an entire host contact network from bacterial MLST data, the distance between a single pair of individuals in the network, defined as the number of nodes in the shortest path connecting the two individuals (the degrees of separation) can be inferred. Intuition suggests that individuals that are closer together in the network would have MLST isolates that are genetically more similar to each other, and simulations from our model confirmed this correlation (Figure S2). Moreover, using the simulated MLST data, we quantified the probability (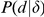) that a given pair of individuals was separated by 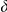 nodes given that the observed allelic distance between their isolates of the commensal bacterium was 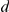 (Figure 3). Given that information, one can then proceed to looking at sets of pairs of individuals. For example, what is the likelihood that individuals A and B are closer together in the network than individuals C and D if the MLST data from isolates from C and D are closer together than the isolates from A and B? In other words, what is the probability that the ordering based on genetic distance of isolates is reversed from the ordering based on network distance? Effectively, this is the probability of type I error, the probability of erroneously classifying the relative strengths of the pairwise distances (between two sets of pairs) in the social network. As seen in Figure S3, the probability for this kind of error decreases as the difference in the number of discordant alleles increases.

## Discussion

One of our primary results, that population genetic data of commensal bacteria can be used to quantitatively characterize social networks of humans, comes at a time of increased focus on the microflora found in and on humans. Already the effort to characterize the human microbiome [14] has had similarly unexpected results, such as the potential use of microbial community composition for forensics purposes [15].

The idea that global characteristics of human contact networks, specifically the degree to which they are structured, embodied by the small world parameter 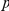, can be inferred from MLST data suggests genotyping of commensal bacteria as a possible tool to quantitatively characterize distinct contact networks. For example, analysis of MLST data might be used by sociologists to rigorously identify differences in social structure between different populations.

Similarly, the result that local properties of host contact networks can be inferred from MLST data, specifically, the likelihood of correctly identifying the relative strengths of links in the network suggests further applications. For example, the degree to which social networks exhibit associate mixing behavior, where there is preferential mixing among certain ethnic, social, and socioeconomic sub-groups of a population, can be quantified by analyzing a subset of the population for a commensal bacterium, running MLST analysis on the isolates, and then investigating whether the isolates from within the different sub-groups are closer together on average than isolates compared across sub-groups.

**Figure 4:**
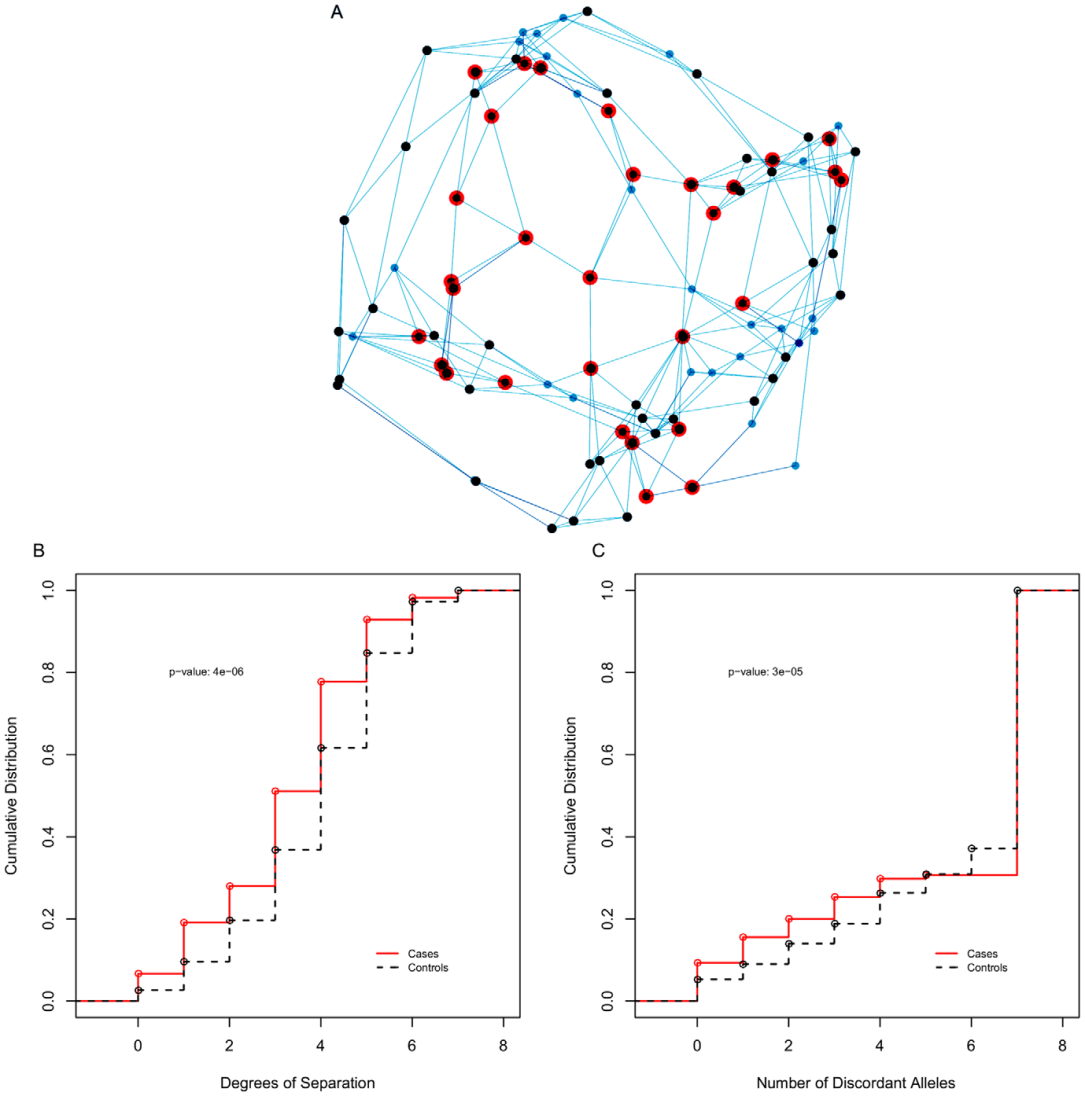
Simulated epidemic on the network. A) Spread of a new pathogen (large red nodes) on a small world network, with an endemic commensal pathogen (small black nodes). B) Cumulative distribution of the network distance for healthy controls in the network (dashed line) and only for individuals infected with the new pathogen (solid line). C) Cumulative distribution of the number of discordant alleles between pairs of isolates from healthy controls (dashed line) and cases (solid line). Both B) and C) show significant differences between cases and controls.

A potential application of this method is the detection of outbreaks of emerging diseases, or the identification of an infectious origin for a disease of unknown etiology. We consider the situation in which an unknown infectious disease is spreading by human to human transmission in a population. Assuming that it is not known whether the disease is caused by an infectious agent (either because it is a new, unidentified emerging disease, or because its infectious origin has not yet been confirmed), we ask, can the fact that this disease is being spread by person to person transmission on the social network be determined by looking only at isolates of commensal bacteria? The methodology would be standard: take isolates of a commensal bacterium from cases and healthy controls, and see whether isolates from cases are closer to each other than isolates from controls. By simulating a disease being spread independently on the same network as the commensal bacterium (Figure 4A), we were able to test this hypothesis. Because the network structure, in particular the degrees of separation between all the nodes was known to us, we first tested whether the distribution of pairwise network distances between cases was different from the distribution of pairwise network distances between controls. The fact that the curve for cases was shifted to the left in Figure 4B is evidence that cases are closer together in the network, which is expected of cases that arise from an infectious disease process that leads to clustering. However, network distance is not generally available in the real world, but we argue that it can be indirectly measured by looking at pairwise distances from MLST analysis of the isolates from cases and controls that happen to be coinfected with the commensal bacterium. Indeed, the distribution of pairwise MLST distances for the isolates from cases was shifted to the left in comparison with the distribution for controls, (Fig. 4C) and this difference was statistically significant, suggesting that it is enough to look at isolates of commensal bacteria to prove that the unidentified disease was spread by person to person contact on the network.

Despite the promising nature of our findings, we emphasize that they are based on simulation results. To rigorously prove our claim that network structure can be inferred from sequence data for commensal bacteria, one would need to validate our method by 1) choosing an appropriate closed population, 2) fully measuring the network structure using existing methodologies such as surveys, 3) isolating and MLST typing an appropriate commensal bacterium (such as *S. aureus*) from the individuals, and 4) testing whether there is correspondence between network distance between pairs in the conventionally measured network and the genetic distance between the pairs of isolates. We propose that future carriage studies of ubiquitous commensal bacteria, in addition to MLST of the isolates, also attempt to measure the social structure of the population being sampled, to test whether MLST data can be used to shed light on the social structures of human populations.

## Materials and Methods

### Simulation of MLST data

We wrote an individual-based model that simulated MLST data for bacteria spread on random small-world networks. First, the model generated a random small-world network using the Watts and Strogatz algorithm [13]. For each individual on the network, the model tracked its state (susceptible/infected) and if infected, the seven MLST alleles of the pathogen. Transmission and neutral evolution of the bacteria were simulated concurrently, with independent events occurring consecutively, according to the Gillespie algorithm. The possible events of the Gillespie algorithm are transmission of infection among susceptible-infected pairs connected in the network with rate 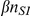, where 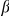 is the transmission rate, and 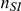 is the number of susceptible-infected pairs in the network; transmission of infection among infected-infected pairs connected in the network with rate 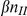, where 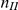 is the number of infected-infected pairs in the network; clearance of carriage (no immunity is assumed and thus the alleles are not under selective pressure) with rate 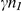, where 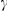 is the recovery rate and 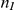 is the number of infecteds; mutation of an allele, occurring with a fixed probability 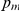 for each transmission event; and recombination, occurring with a fixed probability 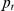 for each transmission from an infected to another infected individual, with the latter two parameters derived from observed mutation versus recombination ratios and observed total mutation rates. The simulations were initially started with a subset of the population infected with a clonal strain. The system was then allowed to evolve, until an endemic equilibrium was reached, marked by a stable distribution of pairwise distances of the MLST alleles from the population. Once equilibrium was reached, the system was allowed to evolve further, and the population sampled at random times to yield simulated MLST data. The process repeated for multiple realizations of the random small-world generator yielded independent observations of MLST data from networks generated with the given parameters.

The number of parameters was kept at a minimum, and can be divided into three categories: the transmission parameters, the pathogen evolution parameters, and the network parameters. The transmission parameters were 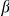 and 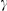; 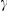 was estimated from the average observed duration of carriage of the pathogen, and 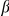 was estimated to fit the observed prevalence of carriage. The pathogen evolution parameters were 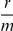, the ratio of the rates of recombination to mutation, and 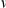, the total rate of per nucleotide substitution. Both of these parameters have traditionally been estimated based on MLST data. The network parameters used to generate the small world network were 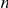, the size of the network, 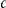, the average number of contacts, and 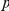, the small world parameter; 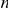 and 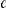 can directly be observed in the field, and we argue that 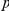 can be estimated from the observed distribution of pairwise MLST distances from isolates drawn from the population.

When choosing parameters for the simulations used to generate the figures, we chose parameters that fit observable data for *S. aureus* (prevalence of carriage, ratio of recombination to mutation, and total rate of mutation). However, we were unable to fit the full model to a real data set from *S. aureus* MLST isolates because important parameters such as the size of network and the average number of contacts are not usually measured and reported when MLST data are uploaded to online repositories.

One key assumption of the model is that the bacteria are assumed to not be under any selective pressure, a potential limitation for bacteria such as *S. pneumoniae* that encounter both vaccines and host immune responses. However, this assumption does not draw away from the main results - that host contact structure can be inferred from MLST data of commensals.

### Inference of Network Structure

To estimate the small world parameter 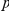, we first generated a table of simulated MLST data for different values of 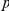. This allowed us to approximate 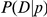 and 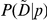, where 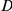 denotes the data - a matrix of pairwise distances - and 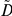 denotes the vector describing the distribution of 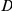.

The posterior probability of 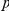 given the network distances 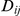 (equivalent to the likelihood when the prior is uniform) was calculated using a variation [16] of Approximate Bayesian Computation (ABC) [17]. Instead of using a cutoff distance as in the original ABC algorithm, the posterior is smoothed using a kernel function. We chose as a summary statistic the empirical distribution of distances 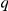


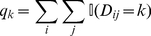
(1)and utilized a Gaussian kernel function
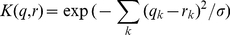
(2)giving the approximate posterior likelihood
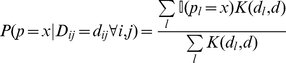
(3)


The value of 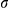 in the kernel was chosen to minimize square error in the posterior mean using cross validation, giving 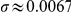.

From the simulations, we can also approximate 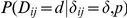, that is, the probability that the number of discordant alleles between isolates from individual 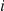 and 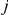 (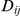) is 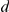, given that individuals 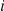 and 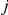 are separated by 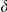 degrees of separation (the number of nodes in the shortest path from individual 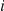 to individual 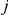) on a small world network with parameter 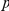. Of greater interest, however, is the posterior distribution 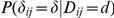, that is, what can we say about the relative connectedness of individual 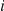 and individual 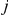 given an observed 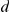 number of discordant alleles in their isolates. This crucial information can be calculated from the simulated MLST data as:
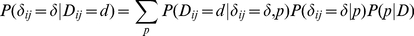
(4)


Here, 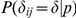 is the expected distribution of degrees of separation in a small-world network with small world parameter 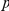, which can be approximated numerically from the simulated runs.

Once 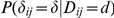 is known, we can calculate the probability that for two sets of pairs in a network, the ordering of genetic versus network distance will be reversed - the probability that a pair of individuals that are closer together in a network than another pair has isolates that are further apart genetically than the other pair:
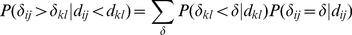
(5)


This equation, calculated for all combinations of 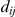 and 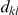, yields Figure 3B.

### Infectious Disease Outbreak Simulations

To simulate an outbreak of a new pathogen, a random individual in the network was infected and the pathogen allowed to independently spread on the same network as the commensal bacterium. Since the infection with the new pathogen is assumed to result in immunity, the outbreak is self-limiting. The outbreak over, the distribution of pairwise network distances of those who were ultimately infected was computed and compared with the distribution for healthy individuals in the network. A chi-squared test yielded a p-value of 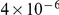, strong evidence that the two distributions were different. The same analysis was repeated, but looking at the distribution of MLST allelic discordance among those coinfected with the unknown pathogen and the commensal bacterium (the cases), and those only infected with the commensal bacterium (the controls). A chi-squared p-value of 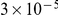 also suggested that the two distributions were significantly different.

## Supporting Information

Figure S1Sensitivity of the shape of the distribution of pairwise number of discordant alleles to key parameters of the model: A) Total per nucleotide mutation rate 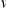, B) rate of recombination to mutation 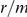, C) number of individuals in the network 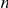, and D) the average number of contacts in the network 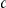. All simulations were run with small world parameter 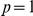 (no local structure), and resulted in distributions either monotonically decreasing or monotonically increasing. The fact that as 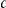, the average number of contacts, goes to 0 this trend is broken, reinforces the result that localized interactions (low 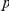) yield the characteristic “U shape”. To generate the figures in the paper, the following parameters were used: 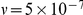, 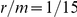, 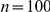, 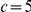, 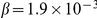, and 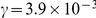.(EPS)

Figure S2 Scatter plot of network distance (degrees of separation) versus allelic difference (number of discordant alleles) for all pairs of nodes in the network. Points are randomly jittered for illustrative purposes. A linear fit to the data (red line) shows a positive correlation between the two distances, and motivates the idea that distances in isolates can be used as a proxy for network distances between individuals.
(TIF)

Figure S3 The type I error for all combinations of observed pairwise distances (see Methods).
(EPS)
