# Supplementary figures and images for: Inferring Social Network Structure from Bacterial Sequence Data
Source: PLoS One. 2011 Aug 1;6(8):e22685. doi: 10.1371/journal.pone.0022685 (PMC3148245; doi:10.1371/journal.pone.0022685)

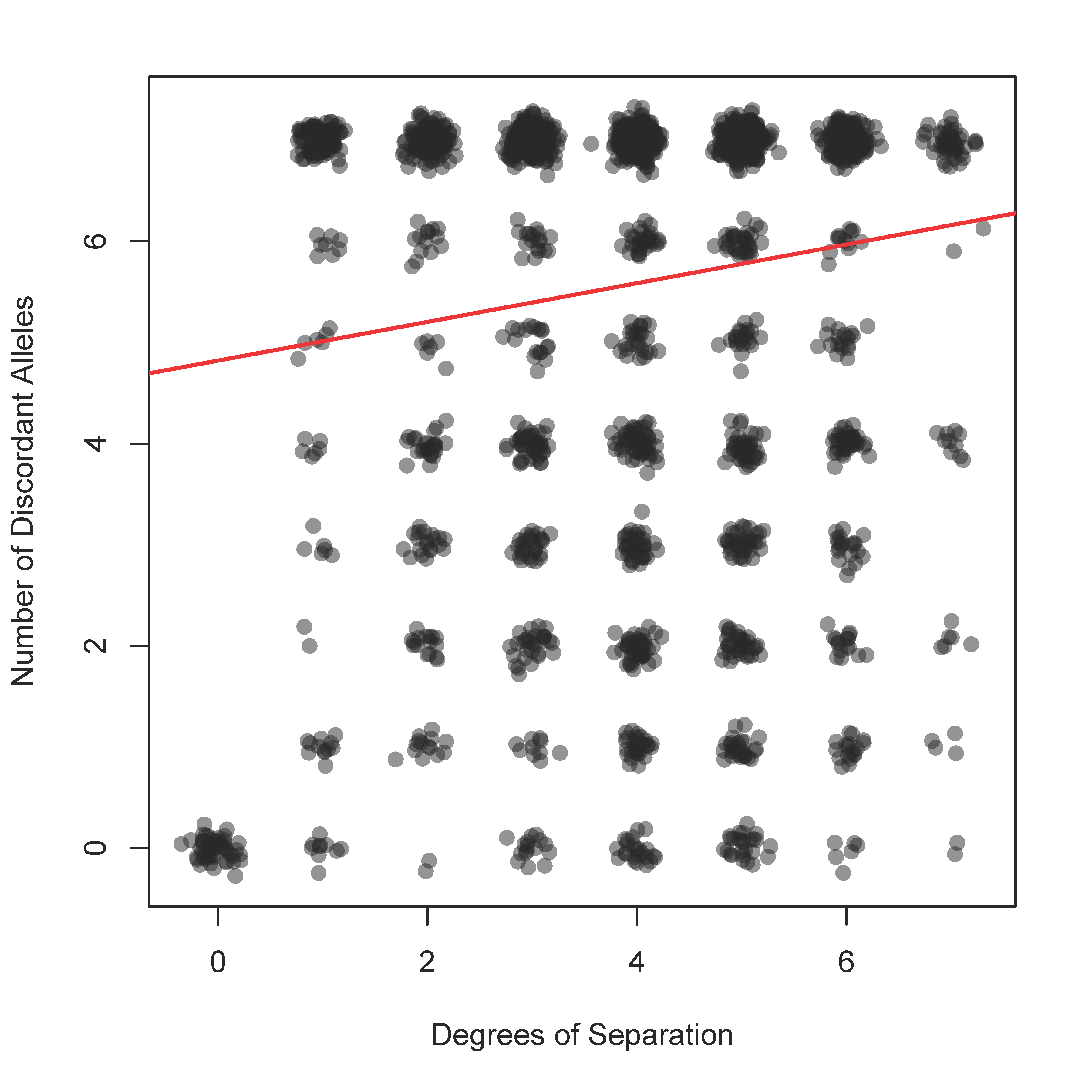

Supplement: Figure S2 — Scatter plot of network distance (degrees of separation) versus allelic difference (number of discordant alleles) for all pairs of nodes in the network. Points are randomly jittered for illustrative purposes. A linear fit to the data (red line) shows a positive correlation between the two distances, and motivates the idea that distances in isolates can be used as a proxy for network distances between individuals. (TIF) [file pone.0022685.s002.tif]
